# S219. RISK FACTORS FOR LOW BONE MINERAL DENSITY IN PATIENTS TAKING ANTIPSYCHOTICS

**DOI:** 10.1093/schbul/sby018.1006

**Published:** 2018-04-01

**Authors:** Min Jhon, Ji-Eun Hong, Cheol Park, Ju-Yeon Lee, Anna Jo, Jae-Min Kim, Il-Seon Shin, Lana Williams, Michael Berk, Jin-Sang Yoon, Sung-Wan Kim

**Affiliations:** 1 Chonnam National University Hospital; 2 Gwangju Mental Health Commission; 3 Gwagnju Veterans Hospital; 4 Chonnam National University Medical School, Gwangju Bukgu Community Mental Health Center; 5 Chonnam National University Medical School; 6 Deakin University, IMPACT Strategic Research Centre, School of Medicine; 7 Deakin University, School of Medicine

## Abstract

**Background:**

The aim of this study is to explore potentially modifiable risk factors for low bone mineral density (BMD) in adults with psychotic disorders. Furthermore, we sought to identify gender-specific risk factors.

**Methods:**

The study included 285 community-dwelling patients with psychotic disorders. Dual-energy x-ray absorptiometry was used to measure BMD. Laboratory examinations included vitamin D and prolactin levels. Low BMD was defined as<1 standard deviation below the mean for young adults. Clinical characteristics associated with low BMD were identified with logistic regression analysis in total population and each gender.

**Results:**

Fifty-eight (20.4%) subjects had low BMD. Low BMD was more common in men and in patients with low body mass indices (BMIs), as well as in those with shorter treatment durations, those on Medicaid, and patients using serotonergic antidepressants. Logistic regression analysis revealed that low BMD was negatively associated with BMI and treatment duration and positively with gender (male) and serotonergic antidepressants use in the overall population. In men, low BMD was associated with treatment duration and BMI; in women, low BMD was associated with BMI, prolactin level, vitamin D, and serotonergic antidepressant use.

**Discussion:**

Low BMI was risk factor for reduced BMD in both genders. Shorter treatment duration was associated with low BMD in men, whereas higher prolactin levels, lower vitamin D, and the use of serotonergic antidepressants were associated with low BMD in women. Psychotropic agents should be prescribed mindful of their effects on bone, as use of these medications is a modifiable risk factor for osteoporosis in women with psychotic disorders.

